# A community-based approach to identifying and prioritising young people’s mental health needs in their local communities

**DOI:** 10.1186/s40900-023-00510-w

**Published:** 2023-11-23

**Authors:** Ediane Santana de Lima, Cristina Preece, Katie Potter, Ellen Goddard, Julian Edbrooke-Childs, Tim Hobbs, Peter Fonagy

**Affiliations:** 1 Dartington Service Design Lab, Buckfastleigh, UK; 2 University College London (UCL), London, UK; 3 Dartington Service Design Lab, Bristol, UK; 4 Dartington Service Design Lab, Hereford, UK; 5 Dartington Service Design Lab, Stoke Gabriel, UK; 6 Dartington Service Design Lab, Bangor, UK; 7 Dartington Service Design Lab, Totnes, UK; 8 Division of Psychology and Language Sciences at University College London (UCL), London, UK; 9 Anna Freud National Centre for Children and Families, London, UK; 10 Child and Family Programme at the Menninger Department of Psychiatry and Behavioural Sciences at Baylor College of Medicine, London, UK

**Keywords:** Community engagement, Participatory research, Community-based participatory research, Young people engagement, Social determinants, Adolescent mental health

## Abstract

**Background:**

Identifying locally relevant and agreed-upon priorities for improving young people’s mental health, aligned with social and environmental factors, is essential for benefiting target communities. This paper describes a participatory approach to engage young people and professionals in identifying such priorities, whilst considering the social determinants related to them.

**Methods:**

We utilised a community-based participatory approach to support young people and professionals in identifying, reviewing, refining, and prioritising, locally relevant opportunity areas that are crucial for understanding and addressing social determinants of young people’s mental health. We adopted a flexible five-stage process, which enabled greater reflection and adaptation in response to young people’s and professionals’ feedback and reflections.

**Results:**

Over seven months, we engaged with young people and professionals in Northern Devon, (a rural area in southwest England), involving over 290 individuals to identify locally relevant priorities for supporting young people’s mental health. Three priorities were identified for subsequent exploration using co-design approaches: (1) identity and belonging; (2) mental health awareness and literacy; and (3) diverse opportunities (for education, employment and leisure). The engagements suggested that designing initiatives and strategies in these areas could contribute to improvements in young people’s mental health.

**Conclusion:**

Young people in Northern Devon prioritised three themes for the next phase of the Kailo Programme—mental health literacy, access to diverse careers and employment opportunities, and identity and belonging within their communities. Rural communities face unique barriers associated with these issues, related to less diverse populations, lack of access to reliable and affordable transport and local industries, and seasonal working. The perceived neglect by authorities towards rural young people has resulted in a lack of activities and opportunities catering to their specific needs, compared to urban areas. Although the government has recognised the need to address these disparities, community members suggest that there is still more work to be done.

**Supplementary Information:**

The online version contains supplementary material available at 10.1186/s40900-023-00510-w.

## Abstract (plain language summary)

The Kailo project aims to help young people and their communities find local solutions for better mental health in the UK. This paper discusses our findings in Northern Devon, England. Through various conversations and workshops with young people and professionals, we identified three key themes: (1) identity and belonging; (2) mental health knowledge and awareness; and (3) a variety of career, education, and leisure opportunities. These themes will be of focus for Kailo’s next phase where ideas and initiatives will be designed and developed with young people and community professionals with the aim to help address important mental health challenges young people are experiencing within Northern Devon.

**Supplementary Information:**

The online version contains supplementary material available at 10.1186/s40900-023-00510-w.

## Background

The delineation between mental health and mental disorders is intricate, and discerning the demarcation between normative challenges and pathological conditions remains an ongoing obstacle within research and practice [1]. While a significant portion of the extant literature emphasises diagnosable mental disorders, mental health may be construed as “*a state of wellbeing in which an individual can realise their potential, cope with the normal stresses of life, work productively and make a contribution to the community*” [2]. Focusing on wellbeing, alongside addressing mental disorders, enables the inclusion of both everyday mental health challenges and diagnosable mental disorders in the lives of young people. This approach underpins the assertion that mental disorders and mental wellbeing are two separate yet interconnected domains, influencing one another through intricate interrelationships [3].

Numerous studies have documented a surge in poor adolescent mental health in the UK [4], with some characterising it as a ‘crisis’ [5, 6]. Evidence also indicates that unmet needs concerning adolescent mental health have persistently troubled experts due to a service-oriented model of care, which fails to account for the developmental trajectory, distinct cultural needs, and appropriate care requirements of young people experiencing mental health difficulties [7, 8]. Support for young people can manifest through formal channels (e.g., healthcare professionals such as doctors, nurses, or psychologists, typically following a diagnosis) or informal avenues (e.g., family, friends, or youth/community workers, irrespective of a diagnosis) [9]. Acknowledging this spectrum enables a more comprehensive examination of the needs and challenges faced by young people concerning their mental health and wellbeing without necessitating a disorder diagnosis, aligning with the aforementioned definitional distinctions. Expanding the concept of support also facilitates a deeper understanding of the pivotal role of local context in the informal support networks accessible to young people, which can significantly impact their overall wellbeing during adolescence. Disregarding these support avenues may result in findings that inadequately represent young people’s authentic experiences [10].

### Young people and social determinants of health

Adolescence represents a crucial life stage [11]. During this time, young people undergo various individual and social transitions, including brain development, cognitive and emotional changes, physical transformations due to puberty, heightened need for social connections, burgeoning interest in romantic relationships with peers, financial independence, self-sufficient living, identity formation, an evolving sense of self, and alterations in their educational environments, such as exam pressures and entering the workforce [12–15]. Consequently, the transitional and developmental nature of adolescence creates an environment in which social determinants can profoundly influence health and wellbeing.

Social and wider determinants of health are terms used within the public health literature to articulate ways that social, environmental and economic factors can affect individual’s overall health and wellbeing. There are various definitions of social determinants of health, the Kailo programme primarily draws from the approaches of the National Institute of Health Research (NIHR) [16, 17] and more specifically Compton et al. [18]. These approaches address the challenges of working at the local community level while incorporating broader social determinants based on national or international research. Other frameworks may not be easily applicable to local community contexts [19], which are the primary focus of this manuscript.

The NIHR approach is useful as it includes 55 determinants of public health across 4 domains; individual, family, community and structural which can be applied in various contexts [16, 17]. However, due to trying to move away from individual characteristics, due to trying to move away from these as social determinants, and focusing more on the community contextual factors influencing young people’s mental health, Kailo has drawn more heavily on the framework of Compton et al. [18], as they provide a useful perspective for examining social determinants at the local level specifically, including, but not limited to, discrimination and social exclusion/isolation, neighbourhood disorder, disarray, or disconnection, and unemployment, underemployment, or job insecurity.

Furthermore, investigating health determinants emphasises the necessity of examining aspects beyond the more commonly explored access to, and utilisation of, healthcare services. By identifying the potential impact of these influential social determinants on young people’s mental health and wellbeing, opportunities arise to examine the resulting implications for the health, policy, and public sectors, as well as to understand the needs of the broader population in terms of resources and support.

### The Kailo framework

Kailo is a research and design programme that seeks to work with local communities, young people, and public service partnerships in comprehending and addressing the social determinants of young people’s mental health. The long-term vision is to create a Framework which can support these communities in improving youth mental health and wellbeing outcomes via the design and implementation of preventative approaches that address contextually relevant social determinants of health [20]. Kailo has three main phases:

1. **“***Early Discovery* including building strong and trusted local relationships, understanding what matters locally, and community forming around shared priorities.
2. *Deeper Discovery and Co-Design* A structured method of youth-centred co-design that takes a systemic, equitable and evidence-informed approach.
3. *Prototyping, Implementation and Testing* A process of embedding designs into local infrastructures and iteratively testing and refining them”. [20]

The initial design and implementation of the framework is occurring in the London borough of Newham and the rural region of Northern Devon in Southwest England. The latter has less densely populated urban areas (the population of Northern Devon is less than half the size of Newham’s), a greater number of hamlets and isolated dwellings, and an older population [21, 22]. Additionally, Northern Devon has some of the least ethnically diverse areas of the UK, whilst Newham is the most diverse area of the UK [23]. These aspects have important implications for the methods used to engage community members. For instance, the cost and logistical challenges of conducting research in small and dispersed areas, with only a small population of young people, and poor mental health outcomes resulting from a lack of knowledge of and access to services [24].

The current study relates to the preliminary research and design phases of Kailo (Early Discovery) in Northern Devon. This involved identifying and prioritising Opportunity Areas (OAs): specific priorities for young people and other professionals (known collectively as ‘community members’), concerning what matters to them in relation to young people’s mental health and wellbeing, and building communities to engage in co-design activities in the next phases of the programme (this differs to the use of the term used within the DofE 2017 report, Unlocking talent, fulfilling potential [25]. Concurrently, a theory-based evaluation is being conducted to “demonstrate the contextual conditions and mechanisms which produce intended (and unintended) outcomes to inform the future development of the Kailo framework” [26].

The theoretical methodology underpinning the Kailo Discovery phase utilises participatory research (PR), an “*umbrella term for a school of approaches that share a core philosophy of inclusivity and of recognizing the value of engaging in the research process (rather than including only as subjects of the research) those who are intended to be the beneficiaries, users, and stakeholders of the research*” [27, p. 326]. It incorporates design and research participatory tools, which are reflective of the needs and resources of the research programme [28].

Central to the Kailo Framework were the principles of community-based participatory research (CBPR). CBPR can guide and inform equal partnerships between professionals (community practitioners, and local and system community leaders who work within the local authority, the local health system, education or locally embedded organisations), young people and research staff, by allowing for more open communication and an appreciation of local knowledge and expertise to address complex problems collaboratively [29, 30]. By seeking to address the power imbalances common within traditional research practices through building trusting working relationships with community members, there is an aim for both young people and professionals to be seen as equal to researchers. This is achieved by acknowledging the relative value of their lived experiential knowledge and experience in surfacing issues and challenges that have a local and population-level relevance [31, 32]. For example, a study by Crane et al. [33] used a CBPR approach to work collaboratively with neurodiverse young people to identify a relevant research topic that reflected on the challenges and experiences of these young people within wider society.

In Kailo, CBPR serves as a basis for genuine, non-tokenistic engagement and involvement of community members in research, enabling them to witness relevant, responsive, and beneficial changes and strategies within their local community. It offers the opportunity to strengthen collaboration and relationship-building strategies, allowing local young people to be regarded as “agents within the system, interacting and influencing the spaces in which they exist” [34, p. 190].

Participatory approaches require trusted relationships across and within the community and with those facilitating the process to promote knowledge and resource sharing [35]. The process must not be burdensome to the community and there must be commitment to community capacity building and equitable knowledge sharing approaches to ensure meaningful involvement; Without organisations and communities having the time and support required to contribute fully, this approach would be inappropriate and potentially harmful [32]. Thus, the Early Discovery phase is focused on involvement and collaboration with community members, to build these relationships and enable the Kailo team and community to engage in subsequent phases which are focused on community collaboration, through co-design and ownership. The Kailo Framework and its phases were designed to create the structures and relationships required to gradually empower the community and shift ownership of the work from Kailo team to the Kailo Community (Local community members) [20]. This phase is focused on breadth of engagement (engaging a wide range and number of community members) to allow more depth (engagement in co-design activities) in future phases Kailo.

## Aim

This paper outlines the methodologies and findings of the programme’s initial research and design engagements in Northern Devon (Torridge and North Devon). The objective was to engage community members residing and working in this local area in identifying specific priorities for supporting young people’s mental health and wellbeing (viewed through a social determinant lens). The central research question posed was: What are the priorities for supporting young people’s mental health from the perspectives of the young people themselves and their local community in Northern Devon?

## Methods

**Fig. 1:**
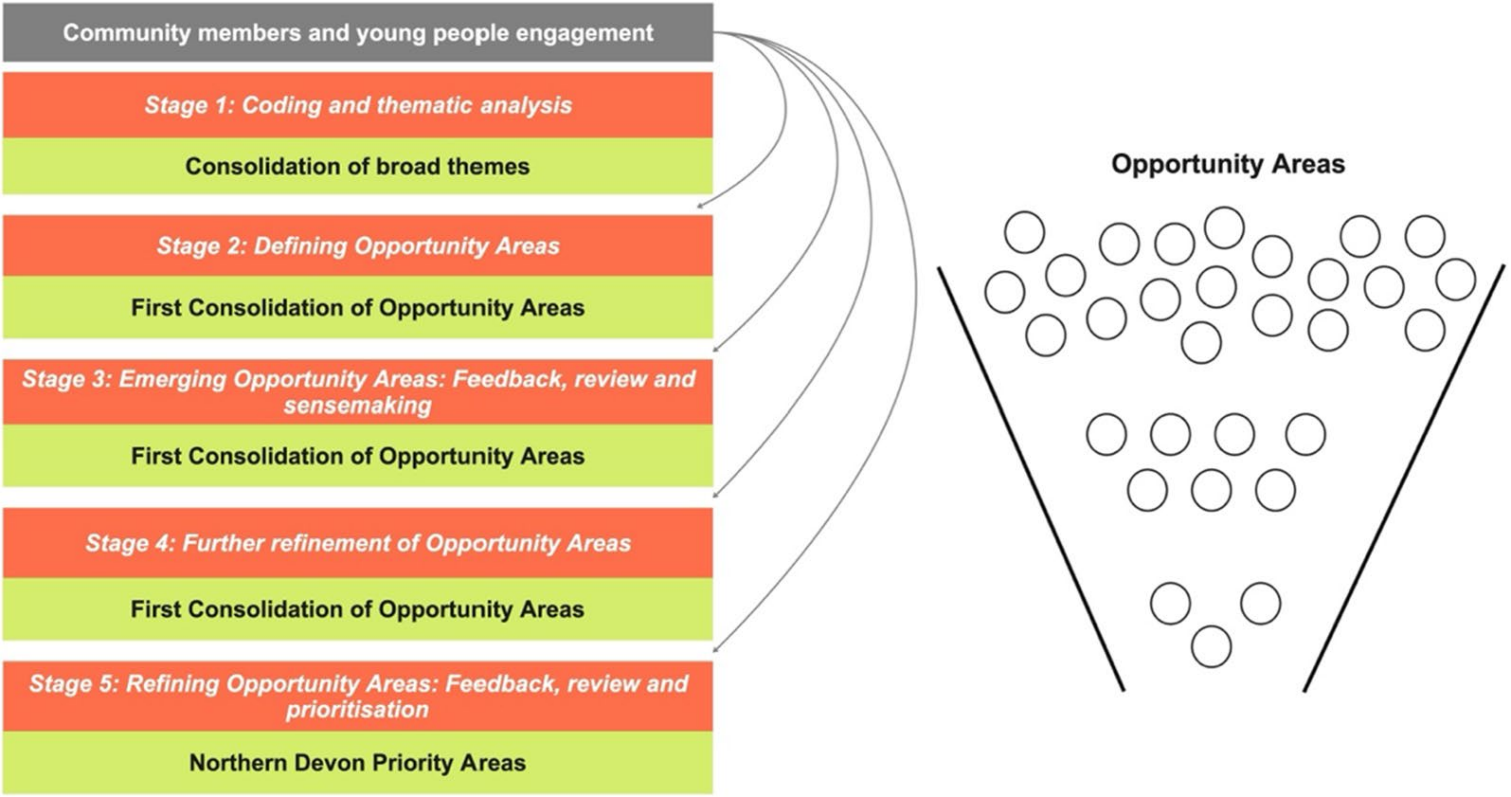
The Kailo Early Discovery Phase Process. The process of refining the broader themes into OAs through the continuous feedback, review and prioritisation from community members living within Northern Devon; Many broad themes are refined over time through feedback, review and prioritisation to become a few key OAs

Kailo Early Discovery phase concentrates on the local exploration, refinement, and prioritisation of specific areas of focus for the development of subsequent strategies, which can be reasonably expected to contribute to improvements in young people’s mental health. To achieve this, in this phase Kailo adopted a participatory research and emergent design approach. This implies (1) the Kailo programme team has utilised the core components of CBPR, while also adapting and refining the methodology based on input from community members, and (2) the changes the programme aims to effect within the community it is currently being implemented in have not been predetermined. By using this approach, Kailo’s particular emphasis on the broad issue of adolescent mental health can be explored and fine-tuned through continuous feedback, review, and prioritisation from community members residing in Northern Devon (Fig. 1).

This process entailed an extended period (May 2022–December 2022) of activities to develop a comprehensive understanding of the local context in which the Kailo programme was to be implemented. This included building relationships with local community members, discerning where needs and opportunities existed, and identifying ongoing local initiatives. This *process was* designed to enable the Kailo team of researchers, designers, public health specialists, professionals with experience of working directly with young people, and most importantly, the young people themselves, to pinpoint locally relevant priorities for supporting young people’s mental health within the context of Northern Devon.

### Setting and participants (including recruitment)

Employing snowball and purposive sampling, professionals were recruited following an initial stakeholder mapping exercise with local systems leaders (Table 1). The inclusion criteria encompassed individuals who either resided or worked in the district of North Devon (and subsequently, Torridge, as recommended by professionals) and who were engaged in initiatives, projects, and organisations aimed at improving outcomes for young people in the area. Engagements included meetings and workshops organised by the Kailo team, and participation in local council, consortiums and towns meetings, fairs and other events.

Young people were engaged through youth organisations and schools, as well as via opportunistic engagements in public spaces in North Devon and Torridge (in key locations suggested by community members). These engagements consisted of short 5–10 min in person conversations in which young people were asked three questions (Table 1), and in person workshops. The criteria for inclusion were age (young people in this study sample were between 12 and 25 years old), and place of residence (young people in this study sample lived in North Devon or Torridge). The Kailo Early Discovery phase approach to engagements were adapted based on community professionals’ insights and learning from Kailo team observations. Thus, to involve a more diverse and greater number of young people in this initial phase, engagements were less structured than the interview guides initially envisioned.

**Table 1:** Topic guides for engagements with community members

5–10 min engagements/conversations with young people	Meetings and workshops with community professionals (1–2 h)	Review sessions Young people (Participation in events organised by schools and community centres. 5–10 min individual conversations with young people)	Review workshops community professional (2–3 h)	Review and Prioritisation Workshops with community members (2–3 h)
(1) What it is like to live in North Devon as a young person? (*Young people responded to these questions through conversations where the Kailo team was taking field notes or through notes that they wrote themselves*)	(1) Engagement with Kailo What resonates about Kailo? What is a red flag or a potential barrier for involvement? Are you enthusiastic about getting involved?	Summary of OAs Clarifying questions General reflections	Summary of the research, adaptations required and responses to feedback Clarifying questions General reflections, suggestions and concerns	Summary of the research, adaptations required and responses to feedback Clarifying questions General reflections, suggestions and concerns
(2) What matters to young people in North Devon?	(2) Existing data sources What existing data is there about YP’s mental health that we should be accessing? How can we access it?	(1) Which ones are more important to you right now? Probing: (I) Why? (II) What does this opportunity make you think of? *Young people responded to these questions through conversations where the Kailo team was taking field notes or through notes that they wrote themselves*	(1) Does this reflect what you told us and what you are seeing in the research? Is this what you told us?	OAs review questions: (1) Why could this be an important area to focus our energy on? (2) Who are the young people and community groups that this topic would particularly impact? (3) Who would be best placed to be involved in responding to this challenge? (4) How are people already participating on this topic?/Whose voice is already being listened to and who’s isn't? (5) What are the risks that we should be aware of when we explore this topic together? (e.g., increasing inequalities)
(3) What wellbeing means to young people in North Devon?	(3) Working with young people Can they introduce us to young people to work with? Which young people do they feel are unheard/undervalued?	(2) Which ones are most important for you? for your well-being?	(2) Is there anything that surprises you about what you are seeing?	Assessing OAs: (1) Enthusiasm and support I have for this OA (2) Impact in young people’s lives in Northern Devon
	(4) What matters to young people? What do they think is important for YP in Newham/North Devon? What is the most influence on their lives	(3) Why have you chosen X OA?	(3) Which of themes resonate with what you see in your roles at work/with young people?	Assessing roles community members want to play for each OA: Roles provided Advocates: *Community advocates would input into the development and design in a light way (e.g., critical friend, cheerleader, supporter, and connector)* Co-Designer *Co-designers would work with other co-designers and Community leaders to develop the vision and take an active role in designing change for the area in focus* Community Leaders *Community leaders would take on a role of convening groups of people to respond to the challenge and support the co-design of the solution*
	(5) Young people’s mental health Do they do any work with YP and communities about mental health? How? Why? What do they think has the biggest impact on YP MH	(4) Is there anything missing?	(4) Reflections around each specific opportunity area	
	(6) Support and infrastructure What currently exists to support YP locally? What works and what doesn’t? What is missing?			
	(7) Kailo Role What resonates about Kailo? What is a red flag or a potential barrier in the project aims and approach?			

In total there were 195 unique engagements (the number of young people) with young people throughout the surfacing priorities stages of Kailo; in addition, 26 young people were involved across various points in the process resulting in 221 non-unique engagements (this is the number of young people engaged across all engagement). Of the unique engagements nearly half (47%) were conducted within public spaces, such as parks and libraries and through ad-hoc street engagements. Of the other half, nearly 40% were conducted within youth centres, and 13% were held within schools. This sample sought to be representative of the wider CYP population in Northern Devon and included young people living in remote rural areas and larger cities, racial minorities, LGBTQIA+, care-experienced, lower-income, and SEND young people.

Prior to engaging with young people, national data and extant literature were reviewed to identify CYP populations more likely to be disproportionately impacted by poor mental health within Northern Devon. These groups included older young people [6, 36], those across gender identities and experiencing various norms and relations [6, 37, 38], those from socially disadvantaged backgrounds [6, 39], care experienced [40, 41], individuals with learning difficulties [42, 43], those with pre-existing mental health problems [44–46], young people living in rural communities [47–49], young people from ethnic and racial minorities [19, 50–52], and young people from the LGBTQIA+ community [16, 53–56]. Engagements with community professionals also focused on identifying under-consulted and vulnerable groups within North Devon and Torridge (e.g., young people in general, those living in remote areas, SEND young people, LGBTQIA+ etc.). From these considerations and discussions, we subsequently sought to actively involve individuals from these groups in the Kailo Early Discovery engagements.

### Data collection

*5–10 min conversations with young people, and initial meetings and workshops with community professionals* Between May and August 2022, individual and group meetings and workshops were conducted with 54 local professionals (11 engaged in the Kailo programme introduction meeting and 43 in subsequent meetings and workshops. See Table 2). These engagements took place both online and in-person across various locations in North Devon and Torridge. All conversations were transcribed and uploaded to an online platform for subsequent analysis.

Between July and August 2022, ad-hoc or opportunistic engagements and workshops were conducted in person with 144 young people at various locations across North Devon and Torridge. While some engagements with young people took place through youth settings, schools and other agencies, the research team also sought opportunities to connect and undertake discovery activities with young people outside of formal service settings in public places like parks, skateparks, shopping centres or other spaces frequented by young people as suggested by community professionals.

*Review, sensemaking and prioritisation conversations and workshops with community members* In addition to the group-specific engagements mentioned earlier, four workshops were conducted with community members between September and November 2022—two online for review and sensemaking and two in-person for prioritisation, attended by a total 76 young people and 109 community professionals. The Kailo team researchers and designers conducted and facilitated all interviews and workshops.

**Table 2:** Timeline of Kailo early discovery activities

Date	Activity	
*Set up*		
Oct–Nov 2021	Project Kick-off and formation of Kailo Workstreams	
Nov–Dec 2021	Initial draft of Kailo Framework	
Jan–April 2022	Plan and set up for engagements with community members	
*Initial engagements: 5–10 min conversations with young people and initial meetings and workshops with community professionals*		
May 2022	Kailo Kick-off events with systems leaders and community professionals in Northern Devon	11 Community members attended
May–Jul 2022	Kailo initiated meetings and workshops with community professionals – Followed by adaptations to young people engagement Identification of local unheard/vulnerable groups of young people in Northern Devon based on conversations with community members and Kailo team observations—Followed by adaptations to young people engagement	Engagements with144 young people 27 conversations with 43 Community professionals
Jul–Aug 2022	Kailo initiated meetings and focus with community professionals Participation in local meetings/events across Northern Devon Engagements with young people	
	Coding and Clustering Early Discovery engagements with community members I (Stage 1: Coding and thematic analysis)	
Aug 2022	Identification of gaps in initial engagements with young people to engage more young people belonging to local unheard/vulnerable groups in Northern Devon—Followed by adaptations in engagements to reach these groups of young people Further meetings and workshops with community members Further engagements and workshops with young people Coding and Clustering Early Discovery engagements with community members II (Stage 1: Coding and thematic analysis) Completion of OA Templates (Stage 2: Defining Opportunity Areas)	
*Review, sensemaking and prioritisation conversations and workshops with community members*		
Sep 2022	Community members Kailo initiated feedback and Sensemaking workshops (Stage 3: Emerging Opportunity Areas: Feedback, review and sensemaking) Kailo team review based on further input from community members (Stage 4: Further refinement of Opportunity Areas)	Engagements with 76 young people (25 of these already engaged in previous conversations) 109 community professionals engaged (most community professionals participated in multiple stages of the Kailo programme)
Oct 2022	Community members Kailo initiated feedback, review and prioritisation workshops Community members feedback, review and prioritisation in local events and meetings Kailo team review and summary of priorities based on community members input (Stage 5: Refining Opportunity Areas: Feedback, review and prioritisation)	
Nov 2022–Jun 2023	Community researchers’ recruitment Kailo formalised partnerships with community organisations	
June 2023–Onwards	Deeper Discovery and Co-design phase	

In this phase of the Kailo programme in Northern Devon, 304 community members were engaged (195 young people and 109 professionals). Many community members were involved in multiple stages of the programme. The methods employed in this research and design programme were approved by the Centre for Social Policy (CSP) ethics committee.

### Analysis

Data analysis in this study was conducted through five stages to facilitate the identification, sensemaking, review and prioritisation of key OAs for young people’s mental health in Northern Devon. Throughout the stages, the number of OAs, and the approach to prioritisation, evolved as the research team responded to reflections, reviews and feedback from community members and engaged in sensemaking and further analysis between these engagements:

#### Stage 1: Coding and thematic analysis

Utilising coding and thematic analysis techniques, the Kailo team analysed field notes and notes gathered from conversations, focus groups, and ad-hoc or opportunistic street engagements. Manual descriptive coding enabled the summarisation of passages from field notes and notes into words or short phrases. From this initial coding broad themes were distilled. A team of three researchers and one service designer conducted the initial analysis, further coding, thematic analysis and review. This stage resulted in the identification of ten broad themes of interest for community members.

#### Stage 2: Defining opportunity areas

In the second stage of analysis, the broad themes and priorities identified during the first stage were reviewed and refined using a structured set of criteria defined by the Kailo Team in an OA template. This stage aimed to ensure that the initially identified OAs were based on community members insights, as well as being aligned with Kailo’s principles of addressing the social determinants of adolescent mental health, were locally relevant to Northern Devon, and were based on extant evidence (empirical and locally identified).

It is essential to emphasise that the Kailo team prioritised the perspectives and priorities of young people over those of professionals. This is because youth voice and agency represent a critical foundational principle underlying the broader strategy development of the Kailo programme. Consequently, while certain themes were significant to the Kailo team, many professionals and some young people, they only became OAs when a substantial number of young people also examined them, and this was made clear to community members. However, during various subsequent workshops centred on reviewing initial analysis and feedback, young people and community professionals predominantly validated each other’s responses.

#### Stage 3: Emerging opportunity areas: feedback, review and sensemaking

During the third stage of analysis, the refined and reviewed OAs (as well as themes omitted) from stage two were shared with community members in Northern Devon to obtain their insights and reflections on the generated research findings. The research team conducted five workshops involving community members (many of whom had previously participated in data collection for stage one). In these workshops, professionals were asked to evaluate the findings’ relevance to their experiences, identify any surprising aspects of the initial analysis and consider whether any elements from interviews and workshops were missing from the first analysis stage. Young people were asked to interpret the surfaced priorities and discuss their subjective importance.

The Kailo research team integrated the feedback from these sessions into their analysis resulting in a final set of 11 OAs, which were reviewed and refined based on further engagements with community members in Northern Devon.

#### Stage 4: Further refinement of opportunity areas

During the fourth stage of analysis, the Kailo research and design team proceeded to refine the 11 OAs identified in stage three of the analysis process. This additional stage of refinement was deemed necessary by the Kailo team, when further examination revealed that some of the OAs from stage three:

- Were solution-focused, prescribing solutions to problems that had not been fully comprehended;
- Represented a cause or consequence of potentially similar broader issues;
- Fell outside the scope of what could be feasibly addressed within Kailo’s timeframe and resources;
- Embodied principles to adopt in the work being undertaken (e.g., services tailored to young people’s needs), rather than pinpointing issues that, if resolved, could reasonably contribute to improvements in young people’s mental health.

As a result of this stage eight OAs were further refined to be prioritised having been identified by community members within Northern Devon as locally relevant, evidence-based and congruent with Kailo’s overarching emphasis on young people’s mental health and wellbeing.

#### Stage 5: Refining opportunity areas: feedback, review and prioritisation

Acknowledging the impossibility of Kailo and members of the Northern Devon community to address all identified OAs, it was crucial to prioritise OAs for the subsequent development of effective and appropriate interventions that are feasible and sustainable in the local context.

Thus, following stage 4, the OAs were re-introduced to community members through workshops and other activities centred on building communities around them using multi-decision-making criteria. These engagements included (1) identifying levels of importance between remaining OAs (community members preferences) (2) alignment with local strategies (One Northern Devon Health Equity Strategy [57] and other local organisation strategies), and (3) sustainability (identifying individuals who could advocate for or allocate resources to future endeavours focused on developing solutions and interventions for specific OAs and prioritising remaining opportunities).

As a result of this stage, three OAs were identified, reviewed, refined and prioritised by community members, as presented in the results section.

Following each stage of analysis, workshops and meetings were conducted with community members in Northern Devon to disseminate analysis interpretations, challenge assumptions, and enhance the accuracy of findings. These also resulted in continuous adaptations to our engagement and analysis approaches (See Additional file 1 GRIPP2 Long Form).

### Reflexivity

**Fig. 2:**
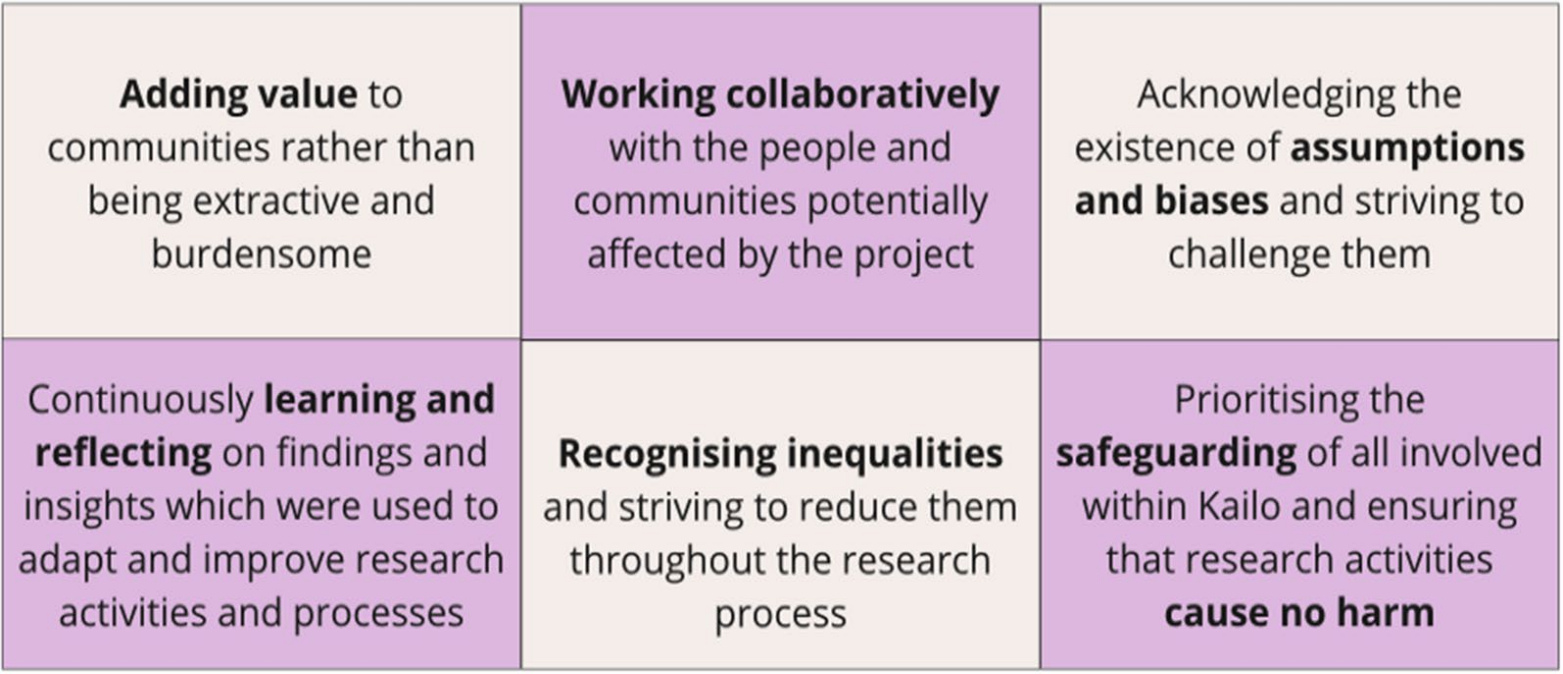
The six foundational principles of Kailo

The following six principles (Fig. 2) were designed by the Kailo team to guide them throughout the research and design process and provide a foundation for ethical and meaningful community engagement. These principles helped the Kailo team aim towards creating a collaborative, inclusive and respectful research space that prioritised the wellbeing of community members.

The Kailo team included a broad range of professionals with experience in adolescent mental health, and belonging to interdisciplinary fields of research, design and practice. The Kailo team that conducted engagements included an experienced social researcher, a service designer, and early career researchers with prior experience of peer research and working with young people with mental disorders. The Kailo team supporting this team included senior academics, public health specialists and strategic designers with experience in using participatory approaches, as well as the community members (See more in Additional file 3 - COREQ Checklist).

Prior to engagements with young people the Kailo team had conversations with relevant community members where the Kailo Programme aims and approaches were introduced, as well as the background of Kailo team members conducting engagements. Community members were able to ask questions and raise concerns, and subsequent adaptations were made to engagements and analysis approaches when needed. The vast majority of Kailo ad-hoc and opportunistic engagements with young people, and all the engagements that took place in formal spaces, were carried out in the presence of a community professional. These professionals were subsequently involved in meetings and workshops where the findings were presented for review.

These iterative cycles of inquiry with community members and involving multiple Kailo team members in the research process served to minimise potential biases and ensured that a wide range of perspectives were considered. These measures contributed to the robustness of the findings and helped ensure that the priorities and concerns of community members were accurately reflected and addressed.

## Results

### Community members participating in early discovery

In total over 101 community professionals, from 45 local community organisations were engaged in Kailo Early Discovery meetings and focus groups sessions. Table 3 outlines community professionals’ organisational types.

**Table 3:** Northern Devon community organisations and professionals recruited and engaged within the Kailo programme

Type	Breakdown of type	Total number of organisations and contacts	Total contacts for type
Youth Organisations	Specialist Support Services (including CYP Mental Health and Physical Wellbeing, and Neurodiversity)	5 organisations (6 contacts)	11 organisations (25 contacts)
	Youth centres/community hubs	3 organisations (10 contacts)	
	Youth and Family Charities (other and non-specialised)	3 organisations (9 contacts)	
Statutory Services and Authorities	Education (including schools, community colleges and academies)	7 schools/colleges (12 contacts)	17 organisations (47 contacts)
	Health Services/Commissioners (including CCG, ICS, primary care, and public and mental health)	6 organisations (27 contacts)	
	Local Government and town councils (including Devon, South Molton, Ilfracombe)	3 councils (7 contacts)	
	Policing	1 organisation (1 contact)	
Funders	Research-based in Health Science	2 funders (2 contacts)	2 organisations (8 contacts)
	Community-based		
Community Organisations (non-youth specific)	Organisations/Charities (including ‘local public services delivery organisations, mental illness and mental health, diversity)	13 organisations (19 contacts)	13 organisations (19 contacts)
Independent practitioners and researchers	Consultants (focused on public health)	(2 contacts)	2 organisations (2 contacts)
Total	45 organisations (101 unique contacts)		

In total 195 young people participated in the Kailo Early Discovery engagements at least once throughout the process. Participants’ ages ranged from 12 to 25 and there was an equal distribution of participants between 12 and 15 (n = 98, 50%, within schools and youth centres where members of staff acted as gatekeepers to support informed consent), and 16 + (n = 97, 50%). Community members highlighted the importance of conducting engagements with young people from different areas of Northern Devon, including rural isolated areas, coastal towns and larger towns. The majority of engagements were carried out within rural areas of Northern Devon such as Howlsworthy, Combe Martin and Martin, and Chittlehampton (n = 107, 55%), with a smaller number conducted in larger towns and coastal areas such as Barnstaple, Braunton, Bideford and Ilfracombe (n = 88, 45%). It’s important to highlight that many of the young people engaged in Barnstaple and Ilfracombe were not residents of those areas but were local to more rural areas around these towns. Community members often requested engagement in diverse locations.

## Findings

### Key themes

**Fig. 3:**
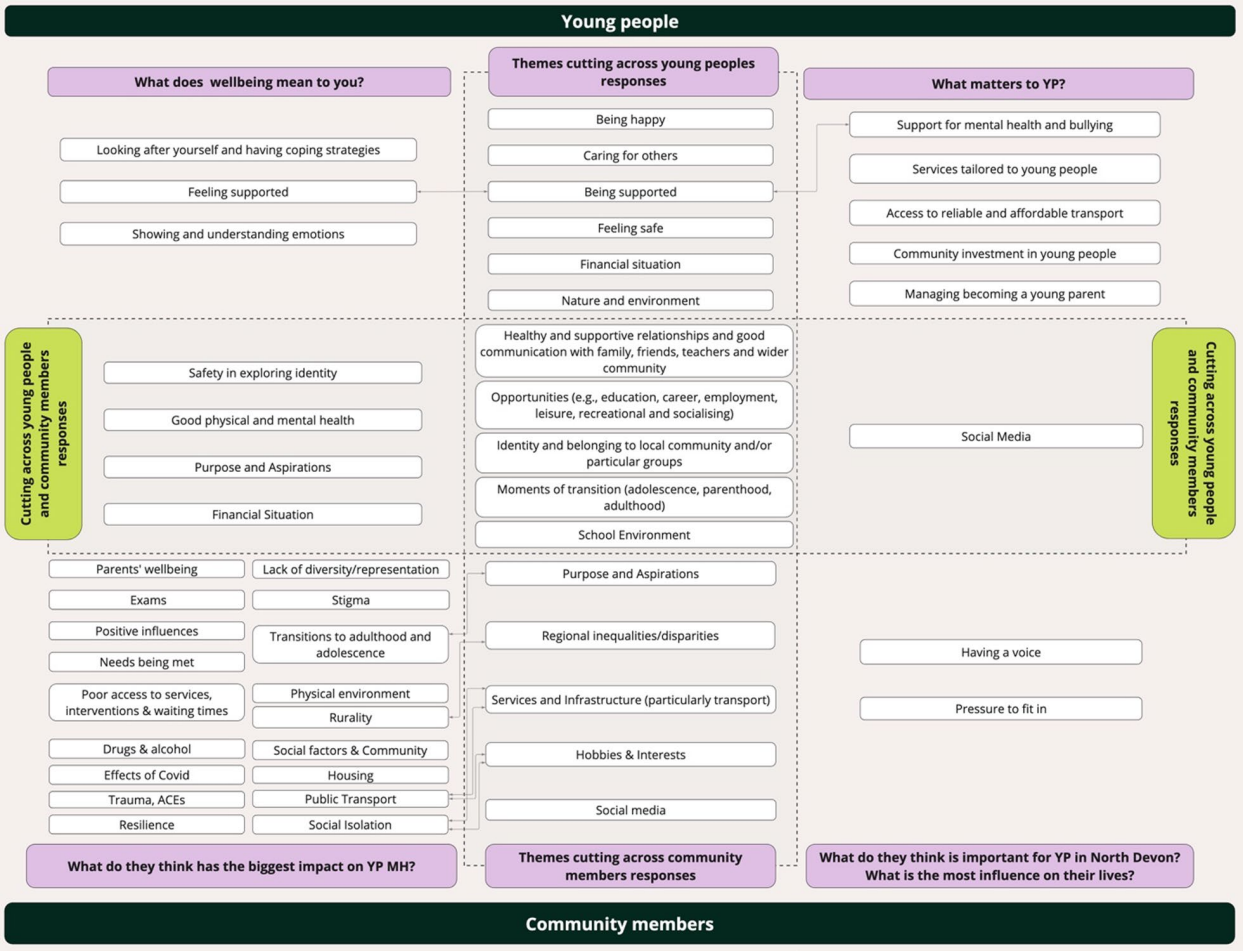
Summary of themes surfaced around what is important for young people’s mental health and wellbeing (feedback from both young people and community members)

Figure 3 shows results from the first stage (See Stage 1 in Methods) of engagement with community members. Themes shared by young people and community professionals when referring to good mental health and what matters for young people locally were clustered to illustrate similarities and differences (See more in Additional file 2 - Detailed Description of themes). The table illustrates the themes that emerged, but not the relative importance of each theme (themes considered more or less important by community members).

These themes, community members responses to other questions, and further exploration of their implications determined the opportunities areas presented below.

### Opportunity areas: feedback, review and sensemaking

The twelve OAs that emerged from the themes explored by community members (See Stage 3 in Methods) reflect the diverse factors that influence young people’s mental health and wellbeing in Northern Devon. These OAs represent areas for potential intervention to address the social determinants of mental health and provide a strong foundation for the development of locally relevant and effective strategies.

### Themes identified only by young people

1. Identity and belonging in their community

**OA:** How might communities be places where young people feel they are accepted, supported, and belong?

Some young people reported experiencing discrimination such as homophobia and racism (“*I get spat at for being gay”; “school is really bad—[there are] homophobes and racism”),* bullying (“*[I*’*ve] been bullied my whole life”*), and stereotyping (community making assumptions about them *“As a gender-fluid person, it is difficult. People keep trying to give you a label”*) as well as a feeling they were being judged by others (talked about a ‘*small town mentality*’ which wasn’t accepting of differences and was unwelcoming of change; *‘So many people don’t accept different identities’*). Young people also reported to finding it challenging to express themselves and gain a sense of belonging because they were unable to access diverse communities or adequate social and emotional support (for example the LGBTQIA+ community; *“Being LGBT*+ *and discovering your identity can be challenging if there is no one else like you around you”*); they felt young people’s mental health and wellbeing was often overlooked or not taken seriously by some in the community (*Feels difficult, people don’t seem to understand’).*

Young people’s responses suggested that by focusing on addressing such issues around social and cultural norms it is possible to create an environment in which young people feel accepted, supported, and valued within their local community. This, in turn, can contribute to improvements in their mental health and overall wellbeing.

2. Exploring rural strengths—the natural environment and rural community

**OA:** Ensuring that young people have equal access to the benefits of the local areas in which they live.

Some young people commented on the importance of features of the local natural environment being accessible (“*Beach is close which is good” and “The moors are amazing to walk on”*), and the unique opportunities present in rural areas (‘there is p*lenty of work in agriculture”*), as well as enjoying the peace and quiet compared to bigger cities (*‘Make things feel better and I can be free’)*. For some, the sense of community with others in the local area was also important, with some young people reporting that they felt safe and comfortable within the area they lived, because they had known other residents for a long time (*‘Lovely community- Comfortable, safe because everyone knows each other”)*.

The findings present a strength-rather than deficit- based approach to an OA which is unique within the overall findings. Analysis suggested that by building on the rural strengths of the local area and introducing young people to the assets of their community and rural living could support young people’s mental health and overall well-being. This approach can help break cycles of disadvantage and empower young people to thrive within their local community.

3. Looking after yourself

**OA:** Creating the conditions for young people to build up a wide range of skills and tools for their ‘wellbeing toolkit’.

Many young people emphasised the importance of having good mental wellbeing (‘*If you don’t look after yourself, it is hard to carry on…to live*) and being able to support their own (Importance of “*Getting my mental health in order. Improving myself as a person”*), as well as their friend’s mental wellbeing (“*Wellbeing of friends matters most to me”*). Some felt that there wasn’t sufficient support available from other sources to build these skills leading them to seek support from peers (such as from schools; “*If struggling with wellbeing talk to friends, schools don’t care after 3 pm”*).

The analysis suggested that developing young people’s emotional literacy so they have the skills and tools to support themselves and their peers with their wellbeing, can further help them better understand, express, and manage their emotions, and develop healthy coping mechanisms. By implementing strategies that actively involve young people, parents, educators, and community professionals, emotional literacy can be cultivated and supported, leading to improved mental health, coping mechanisms, and overall wellbeing for young people.

### Themes identified only by professionals

The following OAs emerged mostly from conversations with professionals. While young people also mentioned family and home life, this was often associated with expewhich will be described below.

1. Parents/Carers and Home Life

**OA:** Improving the quality of family relationships.

Many professionals emphasised the importance of parents/carers and homelife for the mental wellbeing of young people, as it can be these relationships that provide young people with *“trusted figures*” when *“family life is safe and predictable”*. Professionals talked about the difficulties for young people’s wellbeing when these relationships break down or are tested, especially in relation to parent mental health (*“parent stress impacts their young person”*), parenting style (importance of *“putting boundaries in place*”), and family experiences (*“Familial background*” and “*generational influence on behaviour*”, as well as emphasising the importance of providing education so *“parents learn to support their children”.*

Professionals suggested that a family-based approach to mental health awareness and literacy can be beneficial in fostering a supportive environment for young people and reducing barriers to discussing mental health issues. By focusing on parents, caregivers, and the family as a whole, a more comprehensive and supportive approach can be developed to improve mental health awareness, reduce barriers to discussing mental health challenges, and create a positive environment for young people’s emotional wellbeing.

2. Connected systems of support

**OA:** Connecting systems of support to improve consistency and connectedness of services for young people.

Through discussions about service availability for young people, professionals often would note the challenges that comes with the lack of connectivity; there is a *“lack of knowledge about services”, “communities are not connected to local industries”*, there is a *“lack of communication between schemes and initiatives”*. This led to the reflection that there was *“an overarching lack of communication and coordination between systems”* resulting in support “*not reaching young people*”. Professionals suggested that connecting systems of support would result in improved knowledge of available resources, better signposting, and a reduction in inequalities of access. This could support services in providing a holistic approach for young people by allowing the formation of a network of service providers who can communicate effectively; this would help provide the best support for an individual’s needs resulting in better outcomes for young people and their families.

3. Social media usage

**OA:** Increasing education about positive social media use.

Some professionals had concerns about young people’s use of social media and the negative consequences it could have for their mental health and wellbeing. Professionals were concerned about social media exposing young people to *“harmful comparisons”* that “*skew perception and expectations*”, “*inappropriate content*” such as pornography and violence, and the potential link between social media use and mental health problems (“*Things YP (year 6 girls) are watching (online/TV) could be related to increase in self-harm").* However, there was also acknowledgement of some of the positive aspects that social media can provide such a*s* “*using social media to raise awareness and show that you care”*, as well as the “*shift to online interaction has been helpful where access to services was previously limited*”.

By increasing awareness, knowledge and education for all members of the community (including young people, parents and professionals) by highlighting both these positive and more negative aspects of social media, young people would be empowered to use social media in a way that enhances life chances to make informed decisions about their online presence and minimise potential harm to their mental health and well-being.

### Themes identified by all community members (*both* young people and professionals).

1. Nothing to do, nowhere to go in Northern Devon

**OA:** Designing and implementing services tailored to young people.

Young people reflected that they felt there was a lack of services tailored for their age group and needs. This included accessing hobbies (“*Need more projects to join that young people would be interested in”),* organised events* (“events are too expensive or only occur once”),* shops and amenities *(Not much to do, new shops are all for old people)* and places for them to socialise with friends *(Nothing to do for teenagers, all stuff is for kids or pubs for adults).* Further barriers to their access were financial *(There are boxing clubs but nothing else that doesn*’*t cost a lot.),* transportation *(“If you don’t live in town you may be unable to attend some events”)* and the tailoring of the area for older people and tourists *(“Boring for local, but not for tourists*.” and *It is incredibly more tailored for the older generation […] It’s not a place where a young couple would buy a house.”).* Professionals agreed with this, suggesting that young people face a *“Lack of things to do in the summer that don*’*t cost money*”, *‘clubs and activities that are only accessible for some young people”* and that the local area is *“not meeting the needs of young people”.*

The analysis identified a need to provide services tailored to the specific needs of young people that would ensure reducing the feeling, idea and/or experience that there is ‘nothing to do and nowhere to go’. By focusing on providing tailored services and activities that cater to the specific needs and interests of young people, communities can create more engaging and supportive environments.

2. Connection and informal support in close relationships (parents, carers, peers, teachers etc.,)

**OA:** Increasing mental health awareness and literacy to enhance key relationships in young people’s lives.

Some young people suggested that they found it challenging to talk about their mental health with the people around them *(“Having people to talk to is difficult around here”)* as they felt it wasn’t taken seriously enough *(“People overlook YP mental health”)* or the community didn’t have sufficient knowledge or expertise* (“Schools never go full in depth with mental health. People talk about mental health, showing some examples but only have signposting”).* They felt there was a need for more support* (“More support to talk about problems with peers”),* knowing the people they can talk to *(“Everybody has problems, but not everyone is able to talk about them*.”), and being able to be open and honest (“*Being able to say what I need to say and free to do this”).* This reflection was echoed by professionals who cited the importance of young people *“feeling understood”, “being listened to and their views acted on”*, and “*being able to get mental health help when they need it*”.

Analysis suggested that providing a greater awareness of mental health within the local community through literacy could lead to the creation of the conditions, environments and spaces in which young people are able to connect with others, receive informal support, and offer support to their peers.

3. Lack of diverse opportunities/prospects

**OA:** Diversify and increase visibility and access to opportunities available for young people to support them to develop their goals and aspirations.

Young people reflected that they felt there was “*Not a lot of opportunities”, “No different options” and “some good jobs but not many”* in their local area. They also felt that they weren’t being provided with enough opportunities to develop their creativity within school* (“schools should do more creative things not just academic”* and *“there*’*s lack of creative options in education”).* Some young people felt that they would have to look out of area for work *(“I probably will have to travel for work”),* because the industries that they want to gain experience in aren’t available to them easily *(“There are no tech industries to get experience and develop skills”).* There was also reflection on the worries about what others would think of their goals *([I am] worried about people judging my aspirations”),* as well as the insecurity of the local job market and securing full time employment rather than seasonal work *(“it’s difficult to find an all-year-round job”*). Professionals had similar reflections but were especially concerned about job prospects *(“job prospects are really low”),* diversity of choice *(“no industry here for young people”),* and wages* (“lots of low wage jobs”),* and overall, how these impact young people’s aspirations *(“YP are not able to fulfil their aspirations here”*).

Analysis from responses and key themes from both groups suggested that enhancing the quality and visibility of diverse opportunities within Northern Devon can lead to better prospects and outcomes for young people, especially those at a disadvantage. By improving the offer and visibility of diverse opportunities within Northern Devon, young people can benefit from a more inclusive and supportive environment that enables them to access the resources, networks, and experiences needed to thrive.

4. Affordable and reliable transport infrastructure

**OA:** Improve infrastructure and transport to increase access to opportunities and recreational activities.

Many young people mentioned challenges with transport in preventing them from accessing opportunities and activities* (“You can’t do many things if you can’t get from A to B”),* especially the lack of regular and reliable public transport links *(“Not enough transportation in and out of village”; The buses go too slow, there is only 1 every two hours),* the distance they have to travel to reach activities and amenities* (“[You] have to drive 4* + *miles to get to the nearest store”),* reliance on parents and family* (“your parents (need) to drive”*)*, and the cost (“you have to spend money to go places and all the places you want to go to cost money too”).* These difficulties can leave young people feeling *“isolated and far away from all of my friends”,* unable to access opportunities* (“there are not a lot of opportunities because most people can’t drive”)* and a desire to learn to drive* (“what matters to me? Being able to drive and having a car”).* Professionals also echoed this saying* “public transport is a real challenge*” which can limit young people’s access to opportunities *(*“a*ccess to college is a challenge for YP—let alone the social side—because of the transport*”*)* and can be particularly impactful for some young people in particular rural areas (especially “*YP within “small pockets” without transport access*”).

Related to the above OA about diverse opportunities, improvements to transport infrastructure can increase access to diverse opportunities (e.g., studies and employment) and recreational activities, contributing to a more inclusive and connected community in Northern Devon.

5. Appropriate support for young people in moments of transition

**OA:** Creating the conditions for young people to thrive in moments of transition (1) from childhood to adolescence, and (2) adolescence into young adulthood.

1. Adolescence to young adulthood

Some older young people reported struggling with the transition from adolescence into young adulthood with particular emphasis on the challenges of planning for their future (“*Thinking about further education and what you can do after is difficult”),* worries about having to make big life decisions* (“It’s difficult having more responsibilities”),* finances in relation to the cost of housing in Northern Devon *(“I am saving for a house deposit/mortgage. With the rising of prices, the gap between what you save and what you can get with it is increasing”*) and balancing commitments and relationships* (*“*I have that worry and keeping friendships, balancing work and friendships”*). This was reflected in responses from professionals who had seen young people experiencing increased worry around making choices as to whether to leave home to pursue more opportunities than can be offered in their local rural area (professionals have seen *“anxiety around moving to a different area to pursue aspirations*”).

2. Childhood to adolescence

Some young people reported the challenges of transitioning from childhood to adolescence especially in relation to managing physical changes (“*The transition for girls is especially hard because of all the changes to the body”),* not feeling adequately supported (“‘*It can be scary. School doesn’t help enough. (You) don’t know what is going to happen”) and* moving from primary to secondary school* (“you need help with changes at school*”). This was also echoed by some professionals who felt that for young people at this life stage there was the potential for increased worry and a change in relationships and routine in relation to school which could have adverse impacts on mental health (there can be “*anxiety about the transition from primary to secondary school*” and “*the relationship and role of teachers change*”).

Through the initial analysis, it was highlighted that transition periods in rural areas into adolescence or adulthood reflect both more generalised challenges experienced due to the age group (I.e. transition to adolescence and worries about moving up to secondary school) and more rural location specific challenges (having to decide whether to move away from their rural community to attend university or find jobs, or ‘staying’ and potentially being unable to fulfil aspirations) which can impact wellbeing.

By focusing on different age groups, developmental stages and considering the rural specific context to identify challenging times or experiences in moments of transition, then strategies that provide the skills to cope with these times could be developed. This would ensure young people are better supported during challenging transition periods, ultimately improving their mental health and wellbeing in rural areas.

Through further refinement and reflection on the identified OAs, the Kailo team will be able to develop targeted and evidence-based strategies that could address the specific needs and concerns of the Northern Devon community. This process is critical to ensuring that the strategies are locally relevant, effective, and sustainable in supporting young people’s mental health and wellbeing.

### Refining opportunity areas: feedback, review and prioritisation

During feedback, review, and prioritisation sessions (See stage 5 in Methods), noteworthy differences surfaced between the group-specific responses and preferences of young people and community professionals.

Throughout the group-specific feedback and prioritisation sessions, such as those involving only young people or professionals, there were similarities in what both groups considered as the most crucial priority areas to concentrate on, namely identity and belonging, mental health literacy, and diverse opportunities.

Nonetheless, among groups of young people, there was a greater diversity and broader range of expressed priorities: the significance and relevance of all the OAs for mental health and wellbeing were mentioned as top priorities in at least one youth-focused feedback session. However, identity and belonging, mental health literacy, home life, and diverse opportunities were consistently cited as the top priorities across young people’s sessions. This contrasted with professional-only sessions, who predominantly viewed only four of the OAs as most relevant or essential for young people’s mental wellbeing (See Table 4 below).

**Table 4:** Opportunity area prioritisation across group sessions (considering only top four OAs in each session)

Opportunity areas	Feedback, review and prioritisation engagements/sessions (7 in total)			
	Young People feedback sessions (71 young people)	Professional feedback sessions (68 Community professionals)	Community member feedback sessions (23 Young people and professionals)	Total scores for most relevant/important opportunity areas in relation to adolescent mental health
Identity and belonging	10	4	2	16
Mental Health Literacy	10	N/A	5	15
Diverse Opportunities	2	3	3	8
Connected Systems and Support	N/A	2	5	7
Home life	5	1	N/A	6
Transition to adulthood	3	N/A	N/A	3
Transition to adolescence	1	N/A	2	3
Equal access to the benefits of the local area	N/A	N/A	1	1

Table 4 across the seven feedback sessions participants were asked to reflect on which of the OAs presented to them they felt was most important for young people’s mental health. For each feedback session participants' top four of the OAs presented were chosen (these were all given a score of 1 as there wasn’t a distinction in ranking); for some of the feedback sessions, an additional ranking exercise was carried out where participants were asked to rank their four top OAs first to fourth most important (these were given an additional 3 points (overall score of 4) for 1st/most important, an additional 2 points for second most important (overall score of 3), an additional 1 point (overall score of 2) for third most important and no additional points (overall score of 1) for fourth most important)

### The final three prioritised opportunity areas

Following several reformulations, refinements, and reviews (as described in Stages 4 and 5 of the Methods section), the final OAs collectively endorsed by stakeholders in Northern Devon were:

#### Identity and belonging: How can communities become places where young people feel accepted, supported, and a sense of belonging?

- This OA involves creating safer spaces within the local community of Northern Devon where young people feel accepted, free to express themselves, and explore their own identity without fear of judgement or discrimination.

#### Diverse opportunities: How can we inspire, support, and connect young people to access a diverse range of opportunities?

- This OA aims to identify and diversify job and career opportunities for young people in Northern Devon, enabling them to develop, pursue, and achieve their goals and aspirations, while also fostering a sense of purpose and direction.

#### Mental health awareness and literacy: How can we enhance mental health awareness, literacy, and strategies for young people and the wider community to build stronger informal support networks in young people’s lives?

This OA is a result of considerations of three initial themes and opportunities described above: connections and informal support, looking after yourself, and parents/carers and home life.

- It focuses on improving mental health awareness and literacy for young people, their parents/carers, and other professionals, by equipping these groups with knowledge, awareness, and tools for initiating conversations, disclosing issues, and practising self-care. Through this, the community can create an environment where young people can connect with others and receive better quality informal support, including the chance to discuss challenges within trusted relationships.

These OAs will now be the subject of more in-depth research, exploration, and co-design. Members of the Kailo Research Team, and community members will participate in co-design sessions, participatory group system modelling, and evidence reviews to explore and prototype potential pathways for improving young people’s mental health in Northern Devon.

## Discussion

This phase of the Kailo Programme (Early Discovery) explored the key factors influencing young people’s mental health within Northern Devon to identify areas community members would like to focus on in the next phases of the programme (Deeper Discovery). Discussion on findings are presented below as they relate to the research question.

### Identifying specific priorities for supporting young people’s mental health and wellbeing in Northern Devon

Previous research has suggested a deficit in rural research [24], with many UK studies utilising participatory research approaches predominantly focusing on England, with those involving young people mainly conducted in urban areas such as London [58]. This indicates that rural and isolated regions, such as Northern Devon, might be overlooked, which can subsequently impact the representation of the experience of young people residing in these areas within research [58].

The three prioritised OAs which will be advanced to the next phase of Kailo (which will follow a process of co-design), reflect the key priorities for young people living in Northern Devon, and potentially other rural areas, today. Whilst issues related to identity and belonging, lack of diverse study and career opportunities, and mental health literacy are also important for young people in urban contexts [59–62], the barriers and mechanisms surfaced by young people in Northern Devon, which are influencing these outcomes, are more typical of rural contexts. For instance, (1) issues of identity and belonging associated with living in a community that is more homogeneous (e.g., racially, ethically, sexual orientation and gender), isolated and older, (2) limited access to diverse study and career opportunities due to a lack of local industry and role models, the prevalence of seasonal working, and a lack of affordable and reliable transportation, which can lead to young people relocating to urban areas or staying and not being able to fulfil their aspirations [47, 63]. The 2023 study by The Talent Tap and The Aldridge Foundation [64] highlighted the presence of ‘social mobility cold spots’ within rural areas which saw half the young people they surveyed reporting that they didn’t apply to university due to high costs and that they had changed their aspirations for their future careers based on what was available to them locally.

During the Kailo Early Discovery, young people and community professionals identified similar themes and often validated each other’s priority OAs. However, some relevant differences emerged in how specific issues were defined or perceived to influence young people’s experiences and mental health, and in some of the priorities identified. For instance, the Opportunity Area around connected systems of support was surfaced and mostly prioritised by community professionals. A possible explanation for this might be that some young people were initially unaware of the need for these systems to be "connected", or do not perceive this to be one of their most important priorities, in terms of individual needs; young people were explicitly asked about what is personally important to them. Professionals’ responses tended to concentrate on what they (often influenced by their organisational and individual preferences) perceived as the fundamental and structural needs for young people’s health and wellbeing, focusing on the general adolescent age group and structures provided to support them. These differences highlighted the importance of involving different sources of knowledge, and a diverse array of stakeholders in these types of studies [16]. In Kailo this involved ensuring that young people and professionals had an individual voice in defining and prioritising the OAs (rather than always being grouped as ‘community members’). Although connected systems of support were not prioritised by many young people, the importance of bringing this into any strategies developed to improve young people’s mental health as a result of the Kailo Programme was highlighted by young people and community professionals.

### Considering priorities through a social determinant’s lens 

All the OAs prioritised by community members are related to social determinants of health. Whilst literature on social determinants is often focused on national and international perspectives, and frameworks are not easily applicable to local community contexts [65], Northern Devon community members were able to articulate the different manifestations of these determinants in their community. The prioritised opportunity areas are associated with the social determinants of unemployment, discrimination and stigma, relationships and economic inequality (as defined within the NIHR [16, 17] and Compton et al. [18] Frameworks), but translated into local perspectives; For example, community members insights demonstrated how unemployment amongst young people in Northern Devon is related to the lack of access and availability of diverse opportunities in the area. This is a function of not just macro policies, but also local and rural aspects.

Furthermore, many of the OAs can be said to reflect a feeling of lack of investment by authorities into young people in rural areas which perpetuates existing challenges associated with both rural living and these social determinants of mental health; for example, accessing diverse opportunities in a more geographically isolated area is already challenging due to the unreliability of transport services. This could then be exacerbated by a lack of affordable public transport options compounding regional and economic inequalities; young people living in rural areas who are in a better financial position may be able to access more opportunities through being able to access and afford public or private transport costs. This may differ to more urban areas where opportunities may be more geographically accessible and transport more reliable. Recognition of the need to ‘level up’ such disparities between rural and urban areas has been recognised in recent government policies (especially the ‘Levelling up the United Kingdom White Paper [66] but OAs prioritised (and their links to both macro and local aspects) suggest that community members feel more is needed; Thus, strategies to support young people’s mental health should consider both the macro and local manifestations of social determinants in order to be more effective in meeting needs.

### Strengths and limitations

This paper reports findings from the Early Discovery phase of the Kailo programme. In these early stages, the emphasis was on assisting community members in identifying, examining, and refining locally specific and pertinent priorities. The programme successfully engaged 195 young people from diverse backgrounds and needs, as well as over 100 professionals across various sectors (e.g., public health, commissioners, youth work, community charities, mental health practitioners, etc.).

Though the group of young people in this research is diverse, there was a gap in the involvement of some groups of SEND and neurodiverse young people and care experienced young people. However, the adoption of less structured forms (i.e., shorter engagements rather than long semi-structured interviews, and engaging in different spaces) of engagement, as suggested by community professionals, contributed to the inclusion of a wider group of young people in Early Discovery phase. Recruitment for the next phase of Kailo has focused on the identification and involvement of these and wider groups of young people. Adaptations have also been made to support young people who might struggle advocating for themselves by engaging with them individually and in group settings, as well as ensuring young people have the support, they require in future co-design sessions (i.e., having professionals and/or family members present if this is requested by young people). These challenges in participation and recruitment were augmented by the rural and isolated contexts of Northern Devon.

Some community members might have been able to further contribute to analysis and review of research outputs, as well as decision-making if they had been more appropriately compensated. At the start of the Kailo programme, the majority of funding was allocated to the universities, and design and research charities involved. Community organisations were involved through their existing resources and efforts, without any additional resources being allocated for meaningful contributions to the Kailo programme. Lessons from this stage have informed modifications in partnership formation and research funding distribution in the Deeper Discovery phase of Kailo, resulting in increased allocation of funds directly to participating communities and organisations. Community organisations are now Kailo partners with specific funding allocated to their involvement. More resources have also been allocated for involvement of more young people in the Deeper Discovery phase, for longer periods of time. However, not all participation challenges stem from resource distribution issues, as it was already anticipated that in the Early Discovery phase the Kailo team would play a significant role in laying the groundwork for successful co-design in the next phase of the programme (Deeper Discovery).

## Conclusions

Young people in Northern Devon have prioritised three OAs to be taken to the next phase of the Kailo Programme covering the following themes: mental health literacy and awareness within informal support networks, access to diverse careers, employment and leisure opportunities, and identity and belonging within their communities. These OAs cover themes that could also be of concern for young people in more urban areas. However, many of the barriers generating these issues in a rural context, explored by young people in Northern Devon in the Early Discovery phase of Kailo, are more common in rural communities. Young people and community members’ views on what is most important to address for young people in Northern Devon were mostly similar, however, each group provided nuanced ideas of the rationale behind these issues given their unique experiences. Many of the OAs indicate a sense of neglect by authorities towards young people living in rural areas, resulting in a lack of activities and opportunities that cater to their specific needs, which exacerbated inequalities of access to particular opportunities in comparison to more urban areas. While the government has recognized the need to address these disparities, community members suggest that there is still more work to be done.

### Supplementary Information

**Additional file 1.** Completed GRIPP2 Long Form.

**Additional file 2.** Detailed description of themes explored by young people and community members and identified in the coding stage of the Early Discovery.

**Additional file 3.** Completed COREQ Checklist.

## Data Availability

Data sharing is not applicable to this article as no datasets were generated or analysed during the current study.

## Abbreviations

CBPR: Community-based participatory research
CYP: Children and young people
PR: Participatory research
OA: Opportunity area
NIHR: National institute of health research
