# Supplementary material for: A community-based approach to identifying and prioritising young people’s mental health needs in their local communities
Source: Res Involv Engagem. 2023 Nov 23;9:104. doi: 10.1186/s40900-023-00510-w (PMC10666450; doi:10.1186/s40900-023-00510-w)
Supplement: Supplementary file 2 — Additional file 2. Detailed description of themes explored by young people and community members and identified in the coding stage of the Early Discovery. [file 40900_2023_510_MOESM2_ESM.docx]

Supplementary Materials

The present document offers an in-depth exploration of the primary stage of analysis outlined in the ‘A community-based approach to identifying and prioritising young people’s mental health needs in their local communities’ paper. It delves into the specific themes that emerged through the application of coding and thematic analysis techniques, after initial engagements with community members.

# Results from themes analysis

Figure 1 shows some of the results from the first stage of engagement with young people and community members. Both groups were asked several questions, including those listed below. The questions about mental health and wellbeing were slightly different for young people and community members. For example, young people were explicitly asked "what does wellbeing mean to you?" and "what matters to you?", while community members were asked "what do you think has the biggest impact on young people's mental health?" and "what is the most important influence in the lives of Northern Devon young people?". Despite the differences in the questions, there were enough similarities in their overall themes to allow for comparison as both groups alluded to the concept of what is important for adolescent mental health and wellbeing (see Figure 1).

Figure 1: Young people and community organisations engagements surfaced themes.


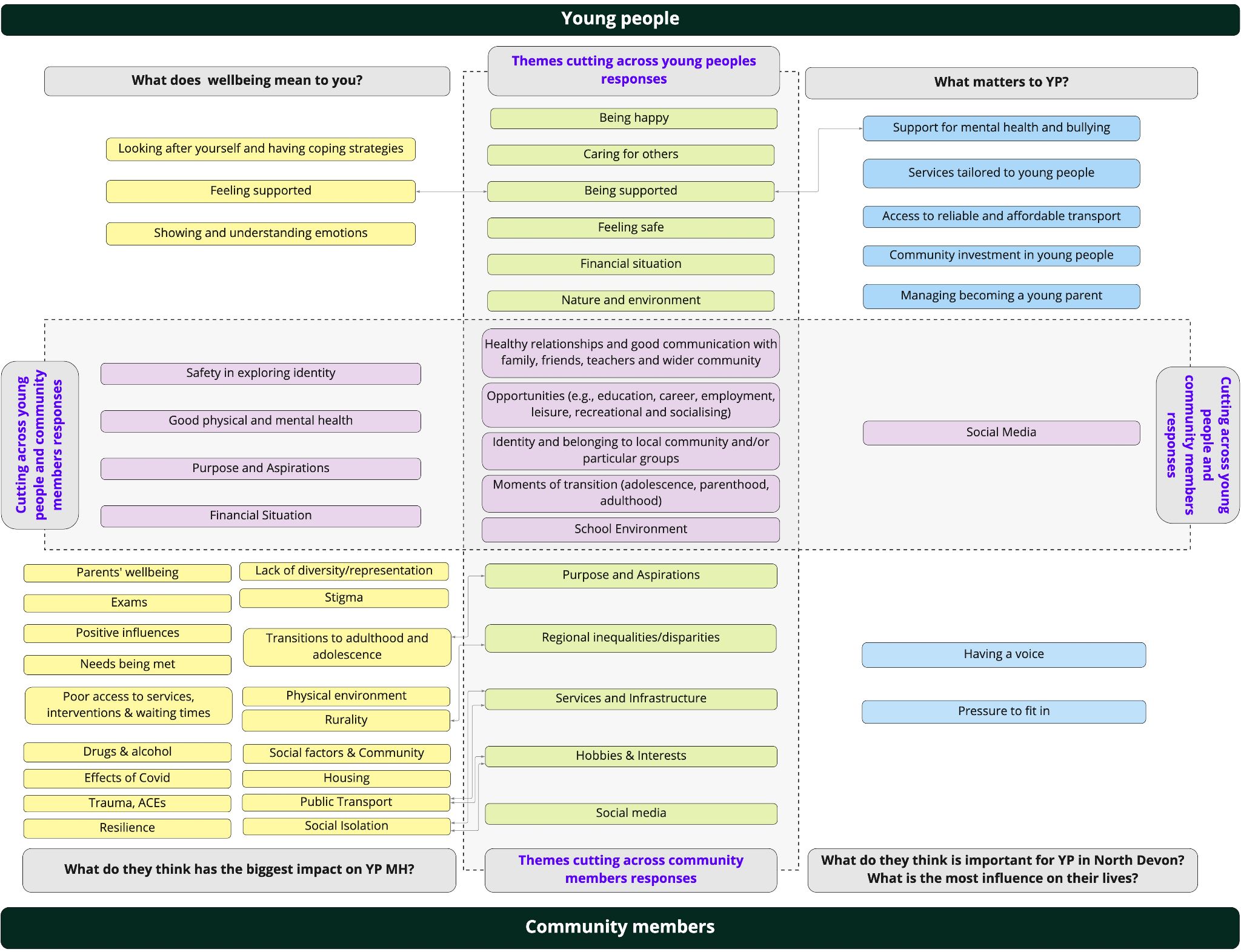


*Figure 1 Summary of themes surfaced around what is important for young people’s mental health and wellbeing (feedback from both young people and community members)*

The findings regarding these questions were mostly consistent with the broader literature exploring mental health, and the social determinants of health, with young people and community members mentioning several aspects of adolescent mental health and wellbeing that are also focused on within the extant literature.

## What wellbeing means to young people and community members within North Devon

**Relationships and communication**

Young people and community members highlighted the importance of having healthy relationships with family, friends, and peer groups. Both groups suggested that having these relationships allow young people to feel supported through communication, sharing and exploration of emotions, identifying needs and managing challenges:

***Family***

Wellbeing was often defined as family, or being with family, as well as being able to ‘talk to them’. Support from parents (or lack thereof) was consistently associated with young people being able to express themselves and be understood in doing so. For instance, young people who felt able to discuss their mental health and gender identity with their parents, identified that the support was more beneficial if they felt their parents were able to understand their needs and challenges. This is supported by [Blum et al (2022](https://www.bmj.com/content/379/bmj-2021-069213.full)) (1) who in their review of the important factors for the promotion of positive wellbeing, health outcomes and development in young people identified the benefits of a strong parent-adolescent connectedness in facilitating the young person to feel able to talk about concerns, ask for advice, and feel supported and cared for. For young people outcomes this sense of connection with family members has been associated with fewer emotional problems, higher self-esteem, increased school achievement, less involvement in violence and antisocial behaviour and less engagement in suicidal and risky behaviours (2).

***Peer groups***

Having friends and belonging to a peer group were both mentioned as important for young people’s mental wellbeing. Some young people also mentioned how unhealthy relationships with peers and bullying have led to feelings of inadequacy and isolation. These finding are supported by the literature, with a systematic review by [Pickering et al (2020)](https://link.springer.com/article/10.1007/s40894-019-00117-x#Sec8) (3) finding that for girls and young women in particular, poor/negative peer acceptance and influence i.e., limited close friendships, negative friendship experiences and relational victimisation, were increased risk factors for experiencing anxiety and social isolation. Moreover, being sociable and able to communicate and explore challenges with someone else was often associated with positive wellbeing outcomes and mental health i.e., engaging with prosocial peers can promote prosocial behaviours in young people (2).

***Self-care and expression***

Young people who are better able to ‘cope’ positively with life stressors are at less risk of mental health challenges than those who have developed ‘maladaptive’ or negative forms of coping (4). The importance of this for young people was reflected in our engagements with many young people and a few community members, who mentioned some form of self-care, often associated with coping strategies and understanding of emotions, as an important driver of wellbeing. Young people defined self-care in various ways, such as the ability to easily access types of places and spaces (e.g., beaches, parks, trails), where one could ‘be themselves’, as well as time and access to opportunities for rest and leisure. Initial conversations suggest some young people can find and access these places and spaces within North Devon, but this was not universal to all, as for some there existed additional barriers such as a need for affordable and reliable transport and financial support.

***Identity***

A sense of belonging within a community or group is important for young people to help form their sense of self and feel safe to explore their identity (5)**.** This was reflected in our engagements with young people and community members who voiced the importance of being able to express one’s identity and feel accepted within their family and wider community for positive wellbeing. Young people were particularly clear about the ability to express ‘who they are’ and ‘feeling like they matter’. This will be further explored in the ‘Context’ section.

***Support in moments of transition***

The report by Viner et al (2012) (2) outlines the importance of the adolescent period for life stage transitions and the changes that can occur from these in terms of increased responsibilities for their health and behaviour, changes to learning and education systems and differences in relationship dynamics. During our engagements, young people and community members often mentioned periods of transition as particularly challenging for local young people’s mental health and wellbeing. These included two crucial stages of transition and the unique shifts and changes that occur within them: transition to teenage years and adulthood.

*Transition to adolescence*

Transition to adolescence was relevant given the changes associated with puberty and the changing nature of relationships, responsibilities and rhythms at home and school. Whilst in transition to adulthood, challenges young people experience are associated with complicated socioeconomic structures in the North Devon area, transition to adolescence is more focused on identity and community. For example, some young people reported issues with being able to express their LGBTQ identity, which could be associated with particular challenges in areas such as North Devon, where a lower percentage of the population identify themselves as members of the queer community (as suggested in statistics from the [Office for National Statistics, 2020) (6)](https://www.ons.gov.uk/peoplepopulationandcommunity/culturalidentity/sexuality/datasets/sexualidentityuk) . These difficulties with living within more rural areas and identifying as LGBTQ+ are further reflected in the findings from the [Government Equalities Office (2019)](https://www.gov.uk/government/publications/national-lgbt-survey-summary-report/national-lgbt-survey-summary-report) (7), who, in their National LGBT survey, highlighted that some people reported moving to larger cities with a more significant LGBTQ population than their hometown due to a greater feeling of safety.

*Transition to adulthood*

In rural areas, transition to adulthood is often followed by young people having to decide whether to move away from their community to attend university and find jobs, or ‘staying’ and being unable to fulfil aspirations. Some of the issues discussed with young people in North Devon included anxiety around making decisions about the future, and a lack of appropriate and diverse local opportunities for studies, employment, and careers. Simultaneously, some young people said they were happy to move away from smaller communities, but felt that they have lost important connections and networks of support as a result.

This outcome is consistent with what might be observed in other rural and isolated communities with low diversity of economic structures (as well as lower-income countries), with young people leading the phenomenon known as ‘brain drain’ i.e. the mobility of skills away from their local rural area and into bigger towns and cities (8). This can result in a lack of diversity and skill competencies within workforces in rural areas, thus discouraging more diverse and highly skilled industries from moving into or staying within the local area (Figure 2). However, this is a simplification of a complex phenomenon, which can be explored in further detail in the next stages of the Kailo Programme.

Figure 2 – Skills and opportunities shortage


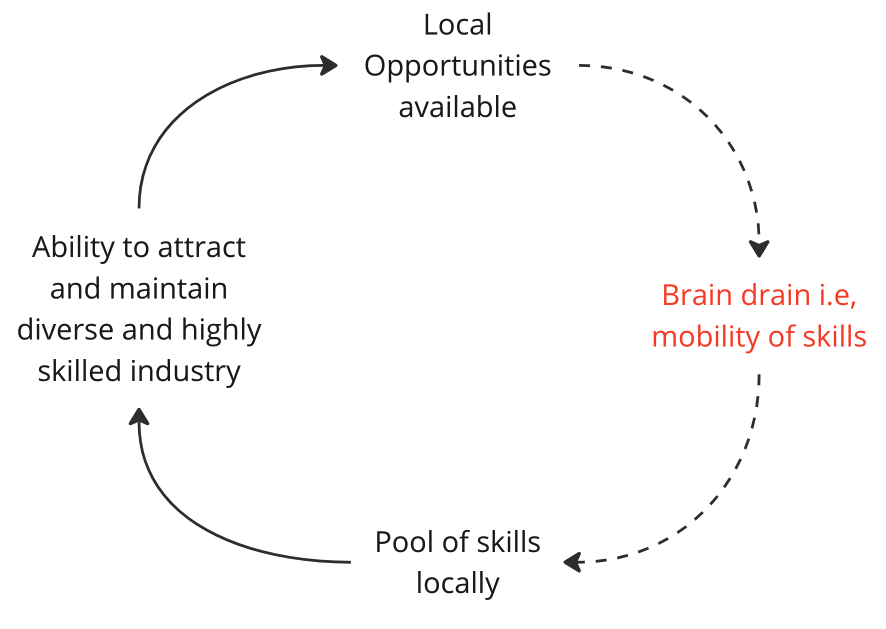


*Figure 2: This figure provides an exploration of potential aspects influencing skills and opportunities shortage in the Northern Devon area*

—-----------

Young people's responses to the question of ‘what wellbeing means to you’ included some themes community members and systems leaders overlooked. Some young people provided responses centred around caring for people in their lives, such as family and friends: ‘caring for others’, helping other people, ‘checking in on friends’, ‘being kind’ and ‘making other people happy’. Most young people also provided responses related to self-care and coping: ‘coping’, ‘sleeping’,’ looking after yourself’, ‘being able to get on with life and struggles’, and ‘having coping mechanisms’. These results indicate the importance young people place on supporting those around them and having the tools and mechanisms to support their own mental health.

After playback and prioritisation sessions with young people and local community members, having the tools and mechanisms to support their own mental health came to be one of the most important priorities for Kailo to pursue in the Deeper Discovery phase. In other words, self-care, which together with better relationships and communication has been translated as mental health literacy, is one of the key local priorities that surfaced through the Kailo Early Discovery process.

## 2. What matters to young people

These included more tangible and external factors often related to the ones already mentioned above. Some of the more different aspects considered included:

***Mental health support***

Young people as well as community members mentioned support around mental health and wellbeing as very important for young people. This response might have been influenced by the fact that interviewees had been introduced to the programme's aims prior to being asked questions about what is most important for young people. However, other engagements and conversations with multiple community members showed that the topic has recently become quite important for young people, and other community members.

**Financial concerns**

Some young people, particularly those between the ages of 18-25 mentioned financial challenges and worries associated with the transition to adulthood, such as having to move away from home for studies and work, anxieties around the concrete and perceived difficulties of purchasing a house, and becoming young parents.

## The Northern Devon Context: Rurality and small communities

Young people and community members were also asked broader questions about the Northern Devon context i.e., community and general infrastructure. Their responses to initial discussions and ad-hoc engagements, as well as their regular participation in feedback, review sensemaking, and prioritisation sessions, provided a rich and complex picture of their Northern Devon communities.

### Benefits of rural communities

Young people, and community members, reflected on what they felt were the good aspects of living within a rural area. For some, this was due to the physical landscape and natural environment, which allowed them to enjoy nature and take part in outdoor activities in their local area. A particular highlight for those living near coastal areas, was being near to or being able to access the beach and sea easily, which young people reflected was a place where they could ‘chill out’ and spend time during the summer.

These reflections also highlighted the social benefits of rural living as some young people's responses indicated that by knowing many of the locals within their town there was a stronger sense of community spirit, people were friendly and they appreciated living close to friends and family. Some young people also compared their local area to bigger cities or more urban areas, suggesting that living within Northern Devon was better as it was more peaceful, relaxed and less stressful. For most, the atmosphere of their local environment was important, with many young people saying they generally felt safe within their town and local area.

### Discrimination in community settings

Young people and community members suggested that the prejudice and discrimination young people experience in their communities may also impact their mental health. This was usually associated with young people’s identities, with young people mentioning various ways their internal or externally represented identities, thinking and expressions of self may be distinct from ‘the norm’ within their local community. These potentially negative experiences may have implications for young people’s ability to express their needs and views, resulting in hiding true expressions of themselves due to a fear of being judged, misunderstood or discriminated against. This can be exacerbated by the size and structure of Northern Devon communities, which due to their lower population density, may result in greater familiarity between community members. For young people, this can mean that expressing their own identities or exploring completely new ones can be a challenging and stigmatising experience, especially if it is a break away from the community norm. As a result, these groups of young people can report feeling unsafe in their communities and unable to be their true selves.

## The Northern Devon Context: Isolation and lack of opportunities and activities

The engagements highlighted mental health and wellbeing challenges particularly relevant for young people living in rural and geographically isolated areas. Rurality, in Northern Devon, was sometimes associated with lack of diverse opportunities and often prospects in one’s local community, which were subsequently associated with challenges in finding employment. Additionally, rural living was associated with challenges around access to affordable and reliable transport, which often led to restrictions in engaging with leisure and entertainment activities and young people’s ability to socialise.

### Nothing to do and nowhere to go (and implications of geographical and social isolation)

A recurrent theme in young people's engagements was a sense that there is ‘nothing to do and nowhere to go’ within Northern Devon. This was also mentioned in discussions with community members who worked with young people. Many young people viewed their local areas as boring and ‘too small’ i.e., lacking shops, events and activities that are tailored specifically to them. Overall, young people discussed the lack of opportunities to socialise, the more critical lack of ‘things to do’ in the winter periods, and in some areas, the focus on activities tailored to summer tourists.

Simultaneously, in some areas, young people suggested there were ‘bright spots’ in terms of activities for young people, such as youth centres, and local sports clubs. However, these were not widespread opportunities and it was suggested that these might be concentrated within bigger Northern Devon towns, and thus may not be accessible for those living in more remote rural areas. This means that for young people in more remote areas, and some smaller towns, additional challenges such as inadequate transport infrastructure and potentially low household income need to be considered. When determining whether or not they are able to access opportunities further afield a lack of these resources can mean that some young people are often excluded from the few opportunities available to them. Furthermore, due to the small number and lack of diversity of opportunities and activities available, some young people reflected that even if they were able to access them, they did not find that they fully catered for all needs and particular groups i.e., neurodivergent young people, non-binary young people, ethnic minorities and young people in low-income households.

### Lack of opportunities

Aspects mentioned above are also reflected in a particular lack of opportunities (e.g., study, career, and employment) in the area. Whilst some young people can find local opportunities which are aligned to their interests (mostly in farming and hospitality), most often struggle. Rural areas such as Northern Devon often lack diverse types and numbers of opportunities for studies and careers, due to smaller population and lower diversity of economic structures. The importance of finding and engaging with suitable opportunities for young people is highlighted in some of their reflections from our engagements; having a purpose, aspirations and a direction in life were all defined as being an important part of wellbeing for many but was highlighted as being particularly of importance for young people over the age of 18. These difficulties aligned with what was highlighted in [the 2020 report from the Centre for Menta](https://www.centreformentalhealth.org.uk/sites/default/files/publication/download/CentreforMH_TheSpaceBetweenUs_Rurality.pdf)l Health (9), which concluded that the lack of diverse opportunities or access to such opportunities within rural areas can negatively impact a young person’s health and wellbeing due to an inability to access resources, safe spaces to socialise with friends, and employment and education choices.

##

## **References**

1.Blum RW, Lai J, Martinez M, Jessee C. Adolescent connectedness: Cornerstone for Health and Wellbeing. BMJ [Internet]. 2022 Oct 27 [cited 2022 Nov 4];379:e069213. Available from: https://www.bmj.com/content/379/bmj-2021-069213.full

2.Viner RM, Ozer EM, Denny S, Marmot M, Resnick M, Fatusi A, et al. Adolescence and the Social Determinants of Health. The Lancet [Internet]. 2012 Apr [cited 2023 Apr 12];379(9826):1641–52. Available from: https://www.thelancet.com/journals/lancet/article/PIIS0140-6736(12)60149-4/fulltext

3.Pickering L, Hadwin JA, Kovshoff H. The Role of Peers in the Development of Social Anxiety in Adolescent Girls: A Systematic Review. Adolescent Research Review [Internet]. 2019 May 16 [cited 2023 Apr 12];5:341–62. Available from: https://link.springer.com/article/10.1007/s40894-019-00117-x#Sec8

4.Compas BE, Jaser SS, Bettis AH, Watson KH, Gruhn MA, Dunbar JP, et al. Coping, Emotion regulation, and Psychopathology in Childhood and adolescence: a meta-analysis and Narrative review. Psychological Bulletin [Internet]. 2017 Sep [cited 2023 Apr 12];143(9):939–91. Available from: https://pubmed.ncbi.nlm.nih.gov/28616996/

5.Best P, Manktelow R, Taylor B. Online communication, Social Media and Adolescent wellbeing: a Systematic Narrative Review. Children and Youth Services Review [Internet]. 2014 Jun [cited 2023 Apr 12];41(41):27–36. Available from: https://www.researchgate.net/profile/Paul-Best-2/publication/260756474_Online_Communication_Social_Media_and_Adolescent_Wellbeing_A_Systematic_Narrative_Review/links/5fb69e10458515b79751b668/Online-Communication-Social-Media-and-Adolescent-Wellbeing-A-Systematic-Narrative-Review.pdf

6.Office for National Statistics. Sexual orientation, UK - Office for National Statistics [Internet]. www.ons.gov.uk. 2022. Available from: https://www.ons.gov.uk/peoplepopulationandcommunity/culturalidentity/sexuality/datasets/sexualidentityuk

7.Government Equalities office. National LGBT Survey: Summary Report [Internet]. GOV.UK. 2018 [cited 2023 Apr 12]. Available from: https://www.gov.uk/government/publications/national-lgbt-survey-summary-report/national-lgbt-survey-summary-report

8.BBC. Wales’ Brain Drain problem: Can It Ever Be fixed? BBC News [Internet]. 2019 Nov 9 [cited 2023 Apr 12]; Available from: https://www.bbc.co.uk/news/uk-wales-50130590

9.Allwood L. The Space between Us | Centre for Mental Health [Internet]. www.centreformentalhealth.org.uk. 2020 [cited 2023 Apr 12]. Available from: https://www.centreformentalhealth.org.uk/publications/space-between-us

‌
